# Supplementary material for: The ERK5/NF-κB signaling pathway targets endometrial cancer proliferation and survival
Source: Cell Mol Life Sci. 2022 Sep 19;79(10):524. doi: 10.1007/s00018-022-04541-6 (PMC9485191; doi:10.1007/s00018-022-04541-6)
Supplement: Supplementary file 1 — Supplementary file1 (PDF 1899 kb) [file 18_2022_4541_MOESM1_ESM.pdf]

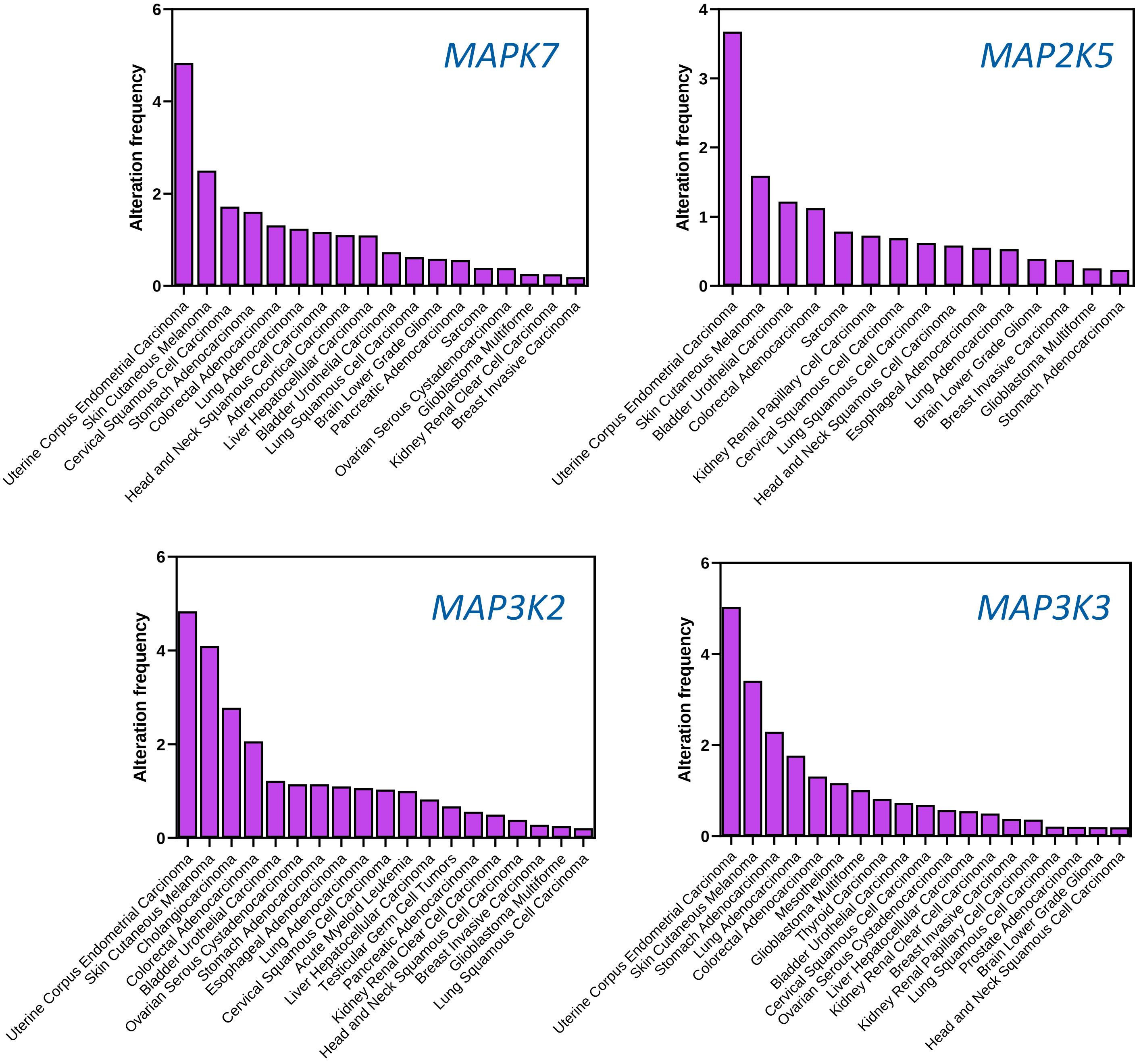

Figure S1. Mutation frequency of the ERK5 signaling components *MAPK7* (ERK5 *gene*), *MAP2K5 (*MEK5 gene), *MAP3K2* and *MAP3K3* in human cancer. Cross-cancer alteration summary from 32 studies from TCGA PanCancer Atlas Studies (https://www.cbioportal.org/)

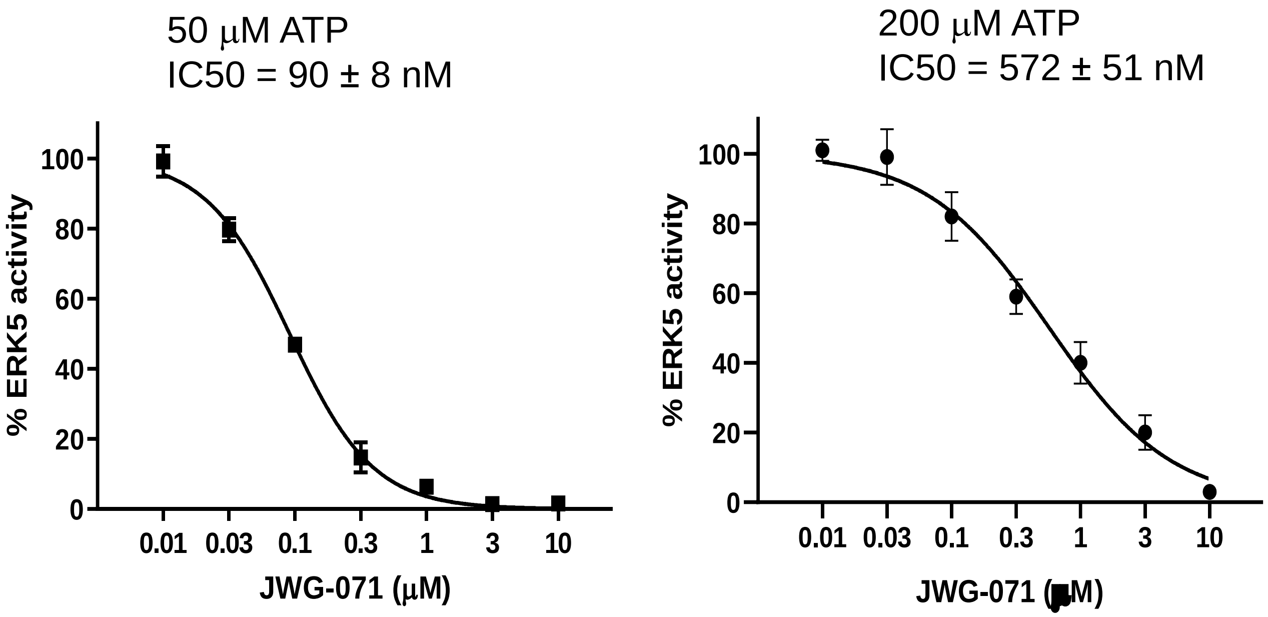

Figure S2. Radiometric assay of ERK5 kinase activity using the ERK5 specific inhibitor JWG-071. ERK5 activity was determined using 200 ng of pure recombinant ERK5, 200 μM PIMtide, and 50 μM (left panel or 200 μM (right panel) ^{32}P-ATP as substrates. Assays were carried out for 20 min at 30°C, terminated by applying the reaction mixture onto p81 paper, and the incorporated radioactivity measured by Cherenkov counting. Data shown were obtained from two separate experiments.

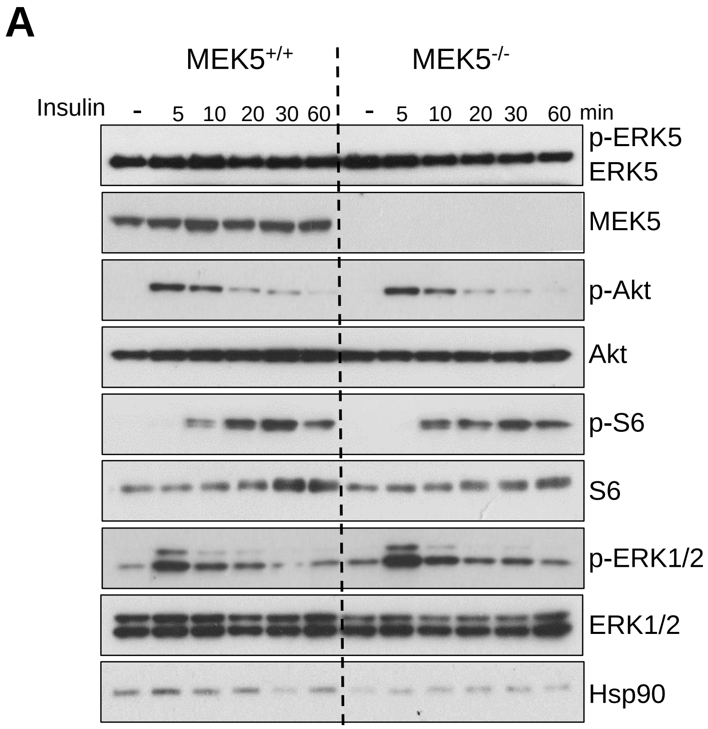

Figure S3. Effect of sorbitol and insulin stimulation on HeLa MEK5$^{+/+}$ and CRISPR/Cas9 MEK5$^{-/-}$ cells. Cells were serum starved, previous stimulation with either 50 ng/ml insulin (A) or 0.5 M sorbitol (B) at the indicated times. Expression of the indicated proteins was monitored by immunoblot. Results are representative of two independent experiments.

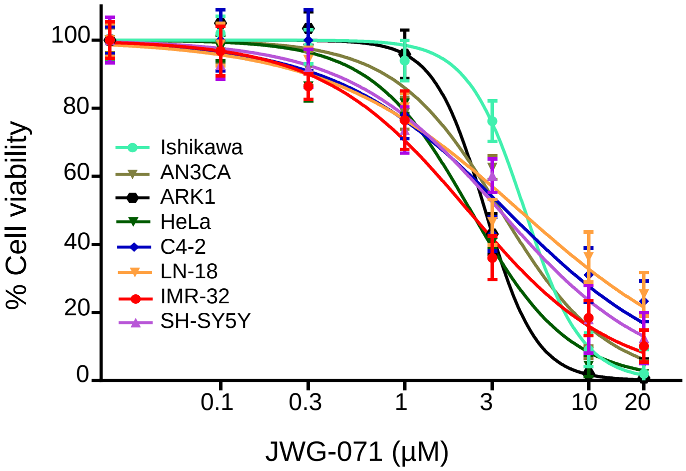

Cell Line	IC_{50} (μM)
Ishikawa (endometrial)	3.2 ± 0.8
AN3CA (endometrial)	3.3 ± 0.6
ARK1 (endometrial)	2.7 ± 0.2
Hela (cervical)	2.9 ± 0.7
C4-2 (prostate)	2.2 ± 0.3
LN-18 (glioblastoma)	2.9 ± 0.4
IMR-32 (neuroblastoma)	1.9 ± 0.3
SH-SY5Y (neuroblastoma)	4.2 ± 1.4

Figure S4. ERK5 inhibition (JWG-071) induces cytotoxicity in a panel of human tumor cell lines. MTT cytotoxicity assay. Cells were incubated with JWG-071 for 48 h. Right table show the corresponding IC_{50} values.

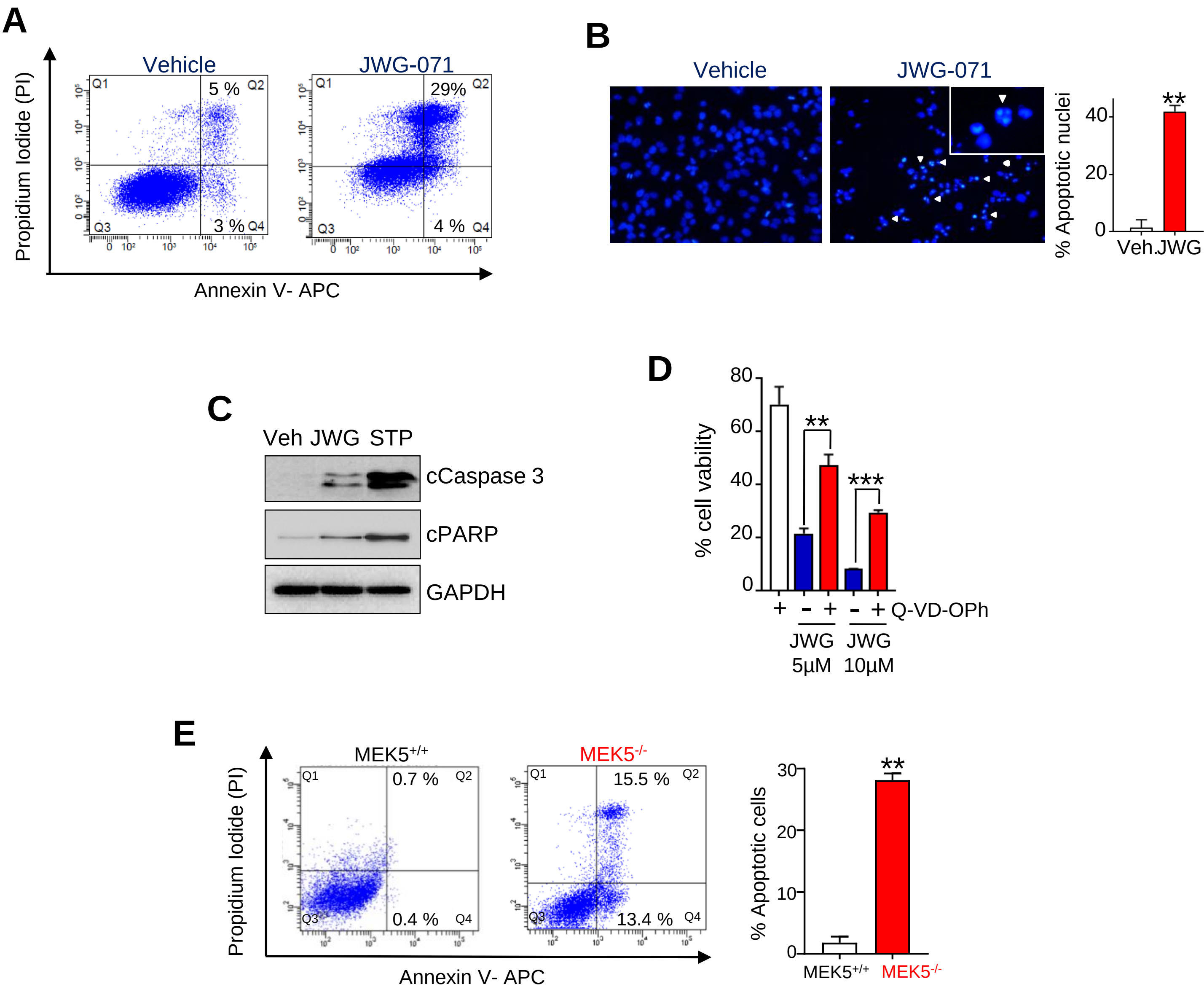

Figure S5. ERK5 inhibition induces apoptotic cell death in HeLa cells. A, Percentage of apoptotic cells was determined by Anexin V/PI staining at 48 h following treatment. Representative flow cytometry plots of cells are shown. **B,** Representative images of nuclear morphology (Hoescht 33258) at 48 h post-treatment with 5 µM JWG-071 or vehicle. Arrowheads point condensed or fragmented nuclei. Right histograms, quantification of the results by scoring four representative fields of each condition (n =3). **C,** Immunoblot analysis of cells treated with vehicle or 5 µM JWG-071 (48 h). Staurosporine (STP, 18 h) was used as an apoptosis control. **D,** Pan caspase inhibitor Q-VD-OPh impairs JWG-071-induced cytotoxicity. Cells were preincubated 1 h with 20 mM Q-VD-Oph, before treatment with JWG-071 for 48 h. Cell viability was monitored by MTT assay. Data is presented as the mean of three independent experiments ± SD, each performed in tetraplicates. **E,** CRISPR/Cas9 MEK5$^{-/-}$ HeLa cells show increased apoptosis, compared to MEK5$^{+/+}$ cells. Flow cytometry assay. Right histograms shows the corresponding quantification of apoptotic cells (Q2 and Q3). **, $P < 0.005$; ***, $P < 0.001$ (Student's t-test).

A

Histological type	Grade	Staging FIGO	Code
Endometrioid	G1	Ia	1
Endometrioid	G1	Ia	12
Endometrioid	G1	Ia	13
Endometrioid	G2	Ia	14
Endometrioid	G2	Ia	2
Endometrioid	G3	IIIc2	3
Endometrioid	G3	Ib	15
Endometrioid	G2	II	4
Endometrioid	G3	Ib	5
Endometrioid	G3	Ia	16
Endometrioid	G2	II	6
Endometrioid	G1	Ia	7
Endometrioid	G2	II	17
Endometrioid	G2	IIIc2	8
Endometrioid	G3	Ia	9
Endometrioid	G3	IVa	10
Endometrioid	G2	Ib	11

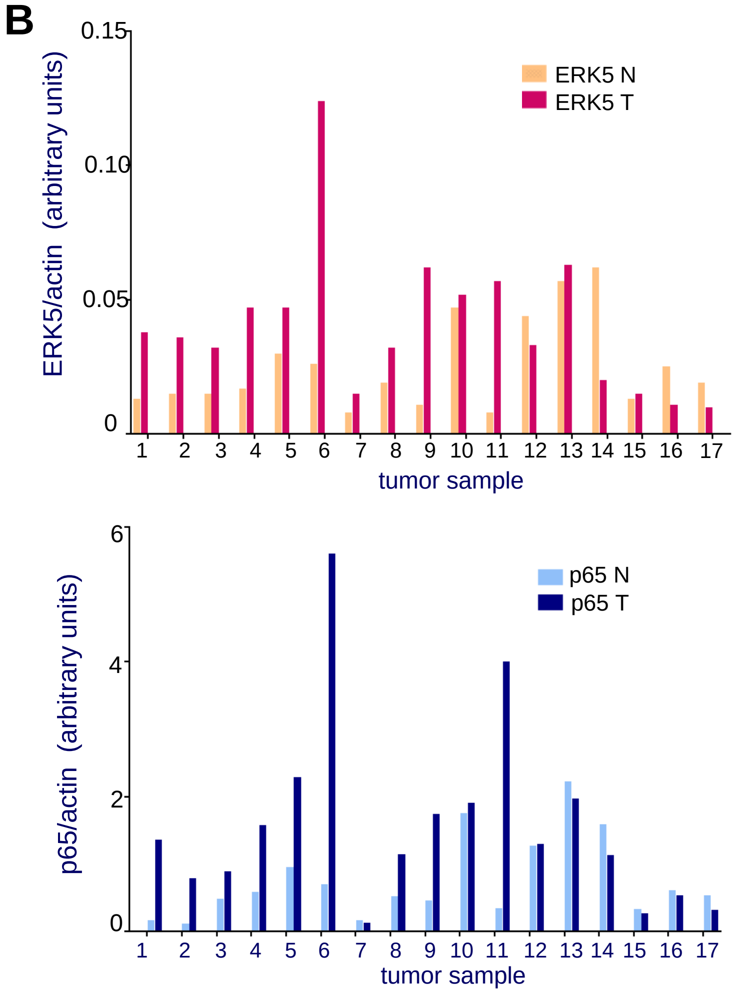

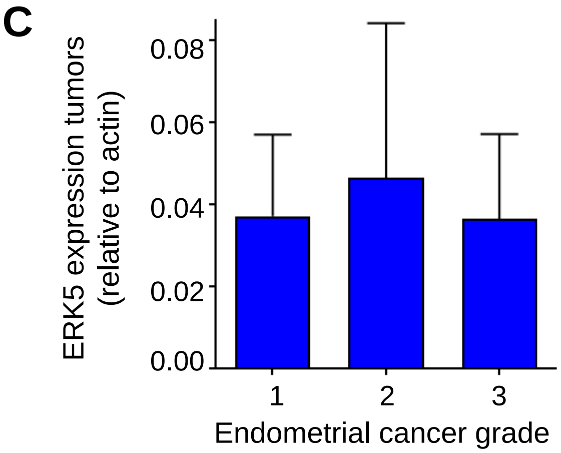

Figure S6. ERK5 and p65 expression in tumoral and non-tumoral samples from 17 endometrioid cancer patients. A, Grade and staging (FIGO) of the 17 endometrioid cancer samples**. B,** Quantification of the ERK5 and p65/RELA protein expression, analyzed by immunoblot signal from Figure 4A. Histograms shows the relative ERK5 or p65/RELA values, relative to actin. T, tumoral; N, peritumoral samples. **C,** ERK5 expression on different grades of malignancy.

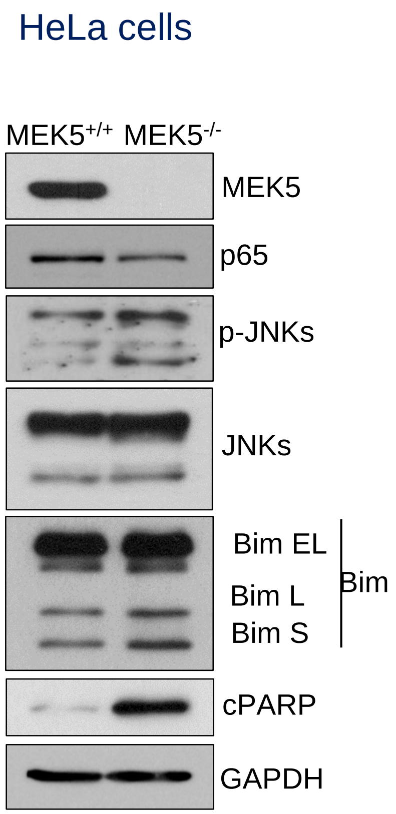

Figure S7. MEK5 genetic deletion in HeLa cells results in impaired p65 levels, and in activation of JNKs and JNK-apoptotic pathway. Protein levels were monitored by immunoblot analysis.

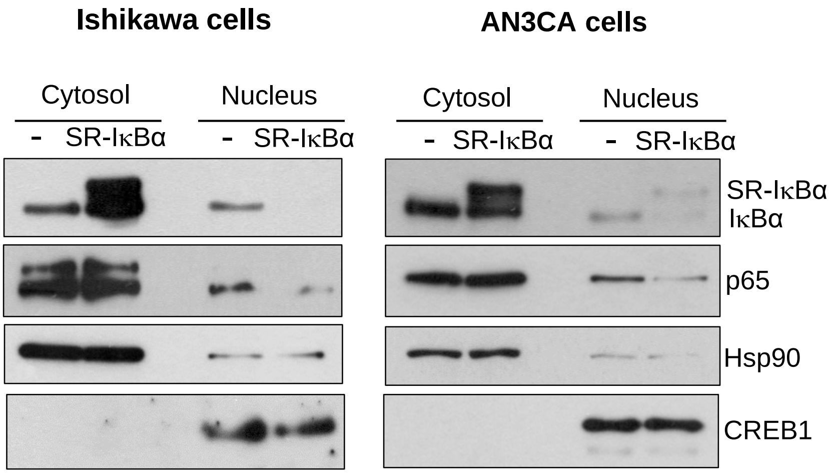

Figure S8. Inhibition of p65 canonical pathway with the super-repressor SR-IκBα impairs p65 nuclear localization. Ishikawa and AN3CA cells were transiently transfected with an empty vector or a vector encoding for a non-degradable IκBα protein (SR-IKBα), as subjected to subcellular fractionation as described in the Materials and Methods Section. Protein expression was monitored by immunoblot analysis.

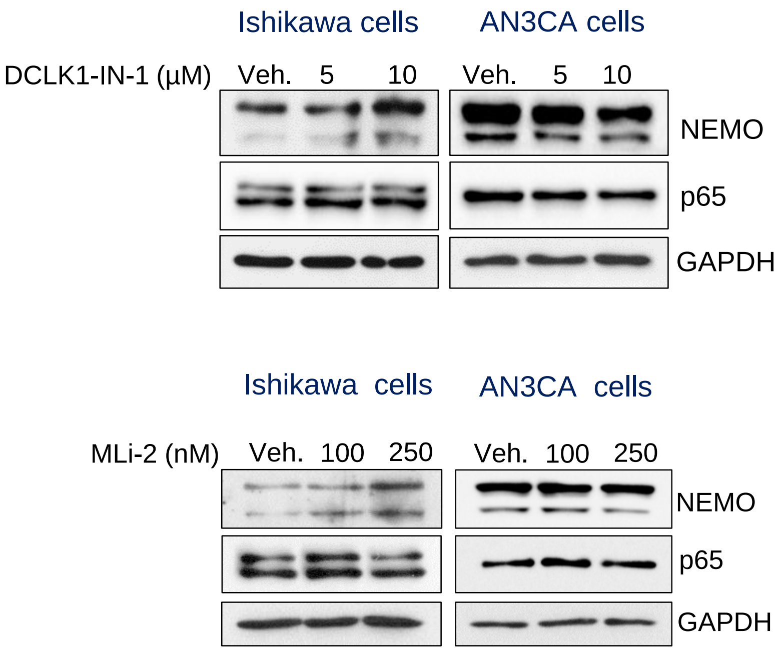

Figure S9. DCLK1 or LRRK2 inhibition does not affect NEMO/IKKγ or p65/RELA protein expression levels. Ishikawa and AN3CA cells were treated with the indicated concentrations of the specific DCLK1 inhibitor DCLK1-IN-1, or the specific LRRK2 inhibitor MLi-2 for 48h. Protein expression was monitored by immunoblot analysis.

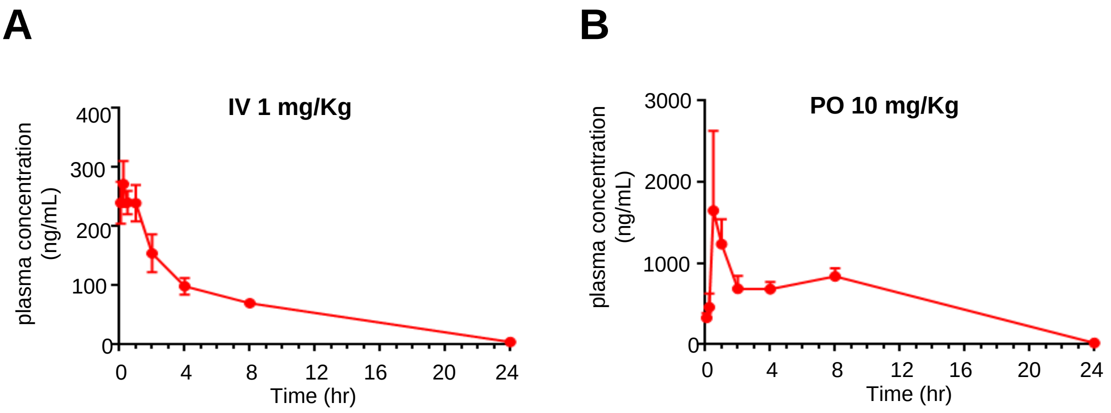

C

Parameter	Unit	IV	PO
Dose	mg·kg^{-1}	1	10
T_{max}	hr	-	1.00
$^{a}C_{o}/C_{max}$	ng mL^{-1}	240.07	1648.76
AUC_{last}	hr*ng mL^{-1}	1632.29	13778.95
AUC_{inf}	hr*ng mL^{-1}	1661.28	13870.05
$T_{1/2}$	hr	4.34	-
CL	mL min^{-1} kg^{-1}	10.03	-
V_{ss}	L kg^{-1}	3.35	-
F^{b}	%	-	84

Figure S10. Pharmacokinetic parameters of JWG-071 (ERK5 inhibidor) in male swiss albino mice. A, Plot of plasma concentration versus time for intravenous (IV) dosing (1 mg/kg), or **(B)** oral (PO) dosing (10 mg/kg). **C,** Summary of pharmacokinetic parameters. Data in A-B are presented as the mean ± S.D. of measurements from n = 9 independent mice assayed per delivery route.

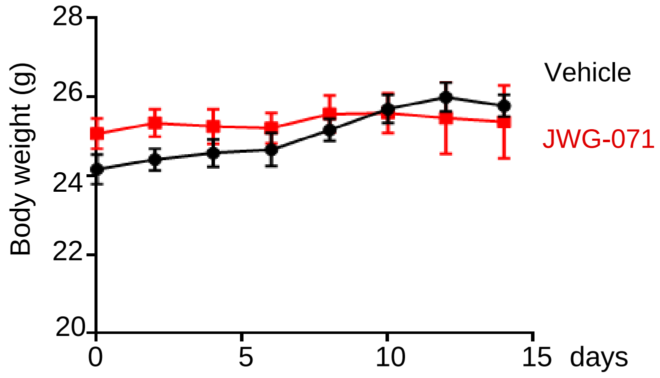

Figure S11A. Mice body weight variation curve in mouse bearing Ishikawa tumor xenografts treated with vehicle or JWG-071 monotherapy. Nude mice were daily treated with either vehicle (black line) or with 50 mg/kg JWG-071 (red line). Mice body weight was monitored every two days.

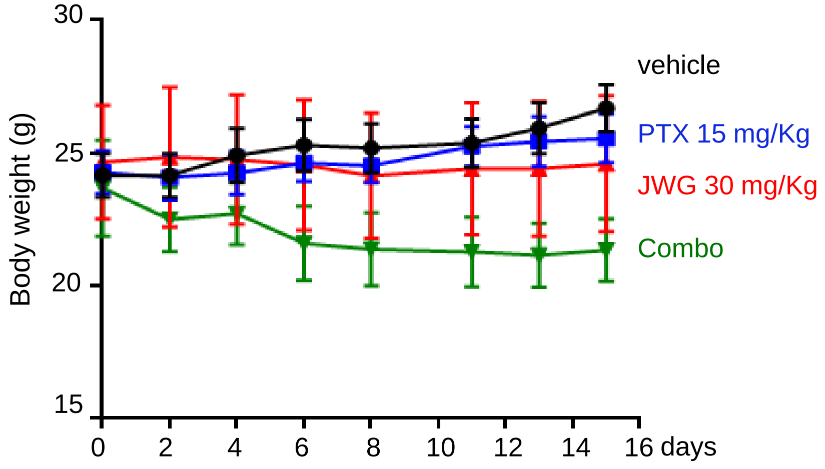

Figure S11B. Mice body weight variation curve. ERK5i in combination with chemotherapy (paclitaxel). Nude mice were treated with either vehicle (black line), 15 mg/kg paclitaxel (twice a week, blue line), with 30 mg/kg JWG-071 (daily, red line), or a combination of paclitaxel and JWG-071. Mice body weight was monitored every two days.

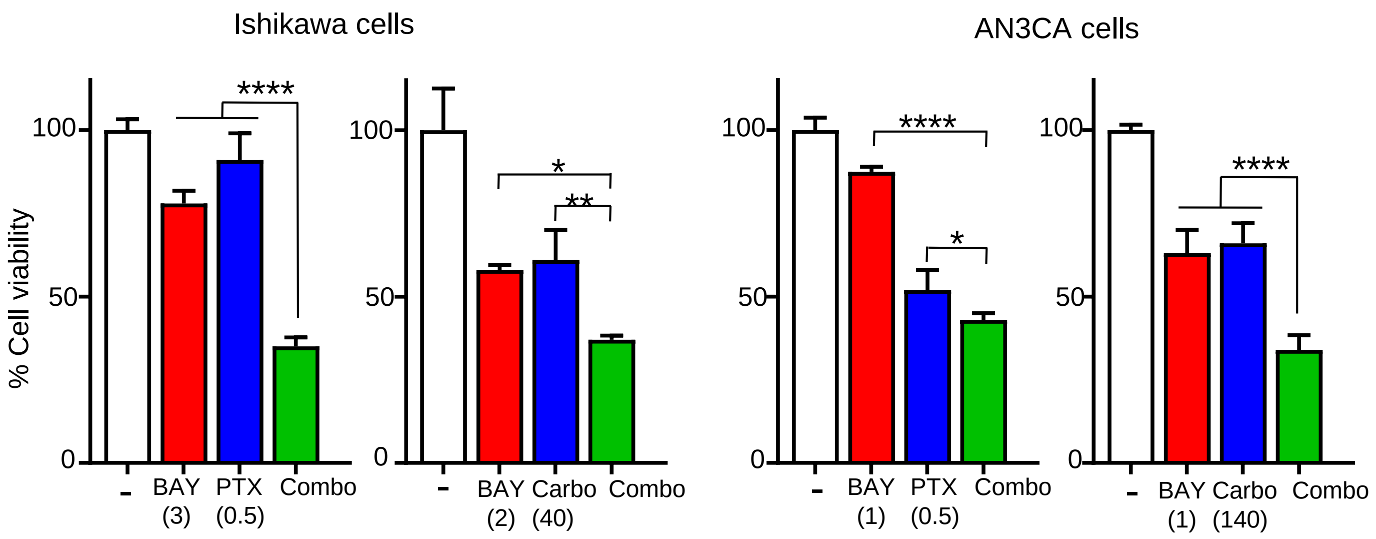

Ishikawa cells

Paclitaxel (nM) \ BAY11-7082 (µM)	0	2	3
0	100	100	78
0.5	100	94	42
1	91	83	35
2	45	53	29

Carboplatin (µM) \ BAY11-7082 (µM)	0	2	3
0	100	100	58
20	100	68	56
30	100	62	56
40	61	42	37

AN3CA cells

Paclitaxel (nM) \ BAY11-7082 (µM)	0	0.5	1
0	100	92	81
0.25	59	68	62
0.5	52	60	44
0.7	40	36	29

Carboplatin (µM) \ BAY11-7082 (µM)	0	0.5	1
0	100	98	73
100	86	80	58
120	78	73	44
140	66	70	34

Figure S12. The NF-κB inhibitor BAY11-7082 sensitizes endometrioid cancer cells to paclitaxel and carboplatin toxicity. Cells were treated with the indicated concentrations of BAY11-7082 (BAY, µM), carboplatin (Carbo, µM) or paclitaxel (PTX, nM) for 48 h, and cell viability was determined by MTT assay. Lower panels: Heat-map analysis of the viability values obtained for the tested inhibitors.

Uterine Corpus Endometrial Carcinoma (TCGA, Firehose Legacy)

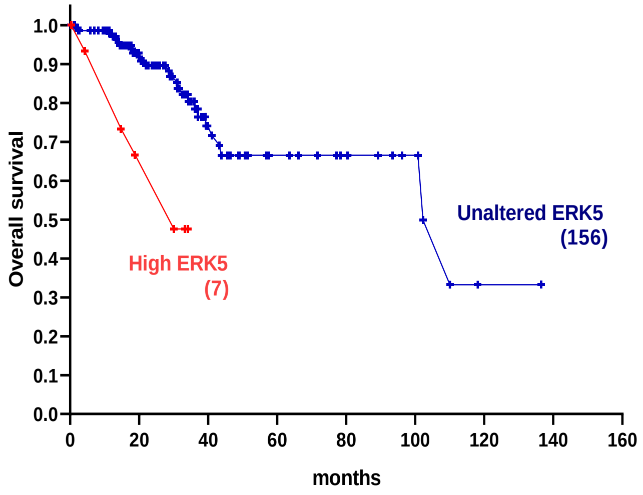

Uterine Corpus Endometrial Carcinoma (TCGA, Nature 2013)

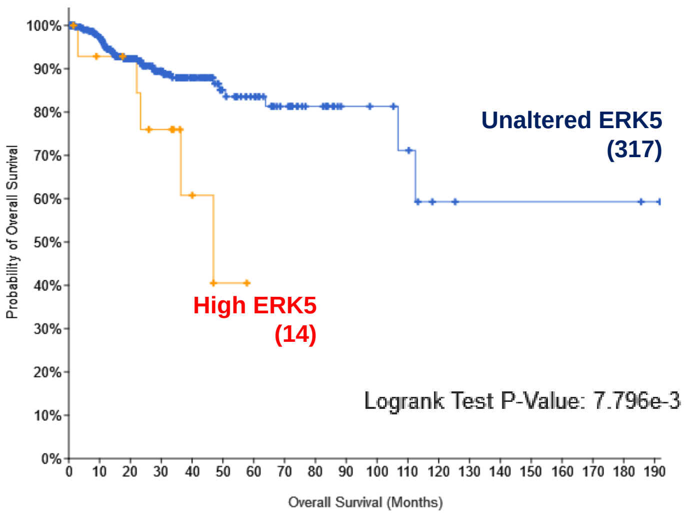

Figure S13. Kaplan-Meier plots of overall survival in patients with endometrial carcinoma. Patients with high (quartile 1) or normal (quartile 2-3) ERK5 mRNA levels are shown in red and blue, respectively. Panels shows the results for the cBioPortal TCGA datasets Firehose Legacy (upper panel) and Nature 2013 (lower panel).

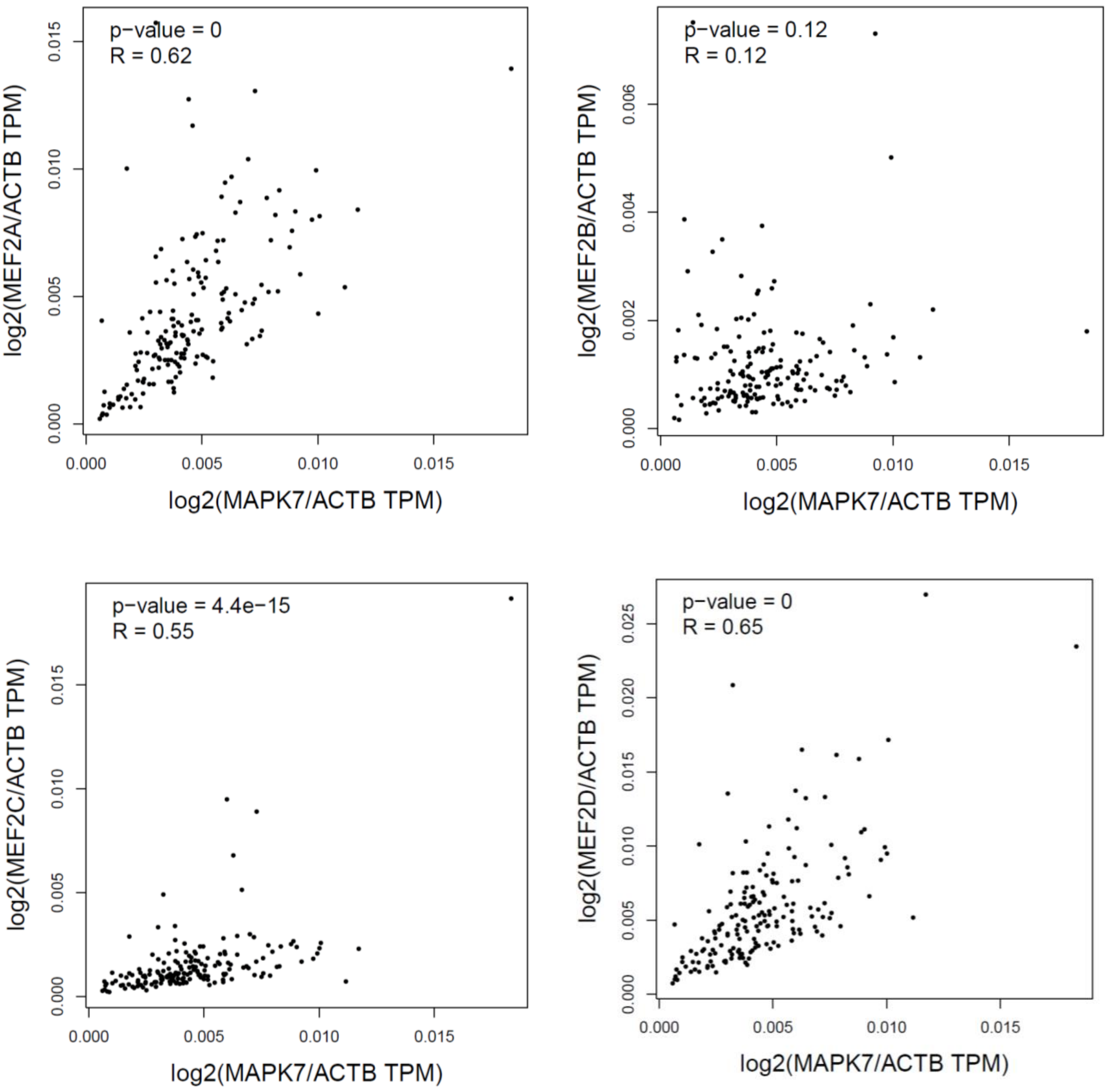

Figure S14. Spearman correlation analysis of ERK5 and MEF2A/C/D mRNA expression levels in uterine corpus endometrial cancer patients. Analysis and plots were obtained from GEPIA website (htpp://gepia.cancer-pku.cn/index.htlm).

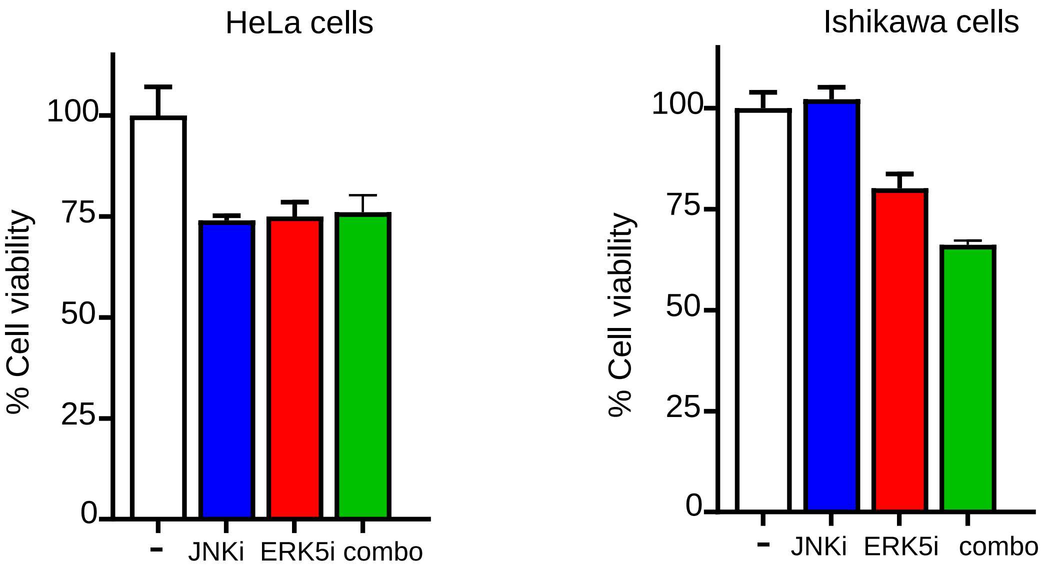

Figure S15. The JNK inhibitor JNK-IN-8 does not rescue the cytotoxicity induced by ERK5 inhibition. Ishikawa or HeLa cells were treated with the indicated concentrations of the JNK inhibitor (JNK-IN-8, JNKi) and/or JWG-071 (ERK5i) for 24 h, and cell viability was determined by MTT assay.

Fig. 2F Tumor growth curves

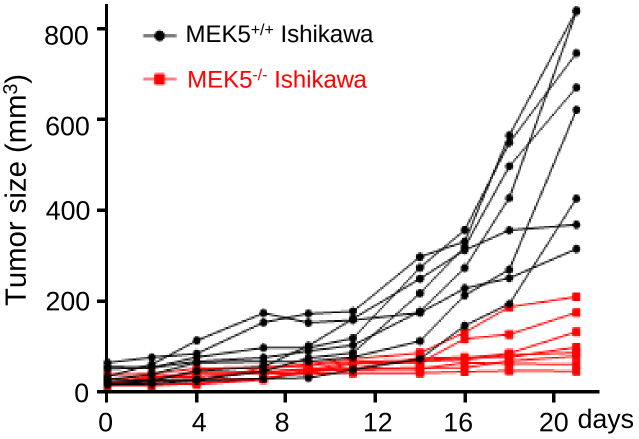

Fig. 6A Tumor growth curves

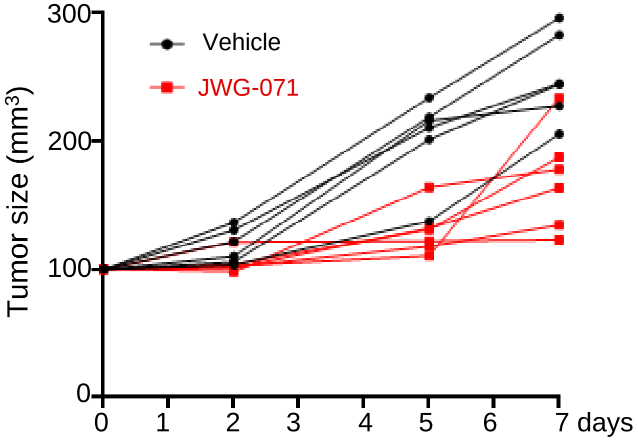

Fig. 6C Tumor growth curves

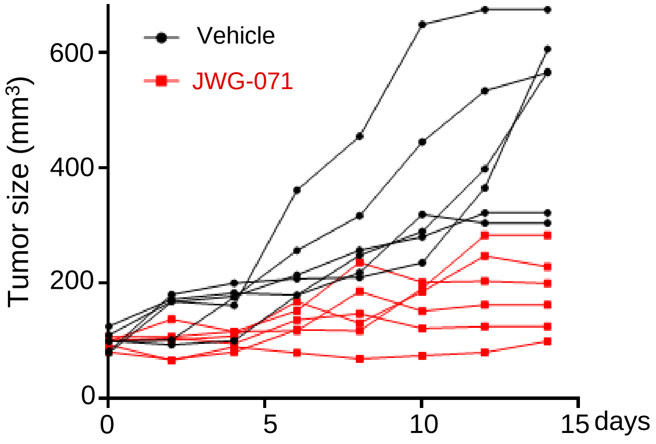

Fig. 7D Tumor growth curves

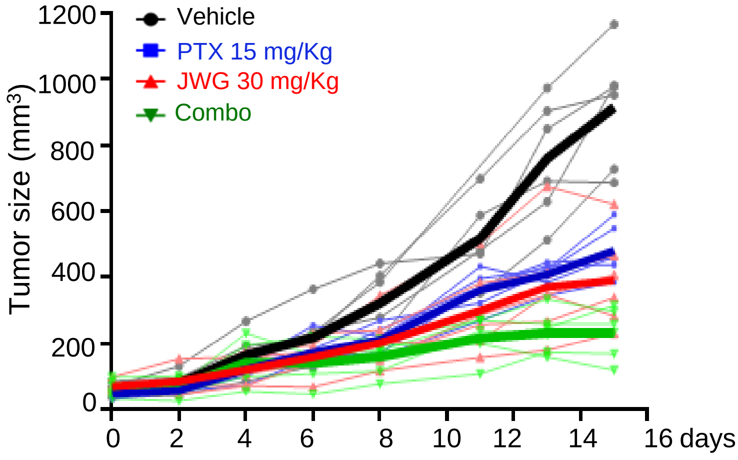

Figure S16. Individual tumor growth curves corresponding to the *in vivo* experiment in mice.
