# Supplementary material for: The ERK5/NF-κB signaling pathway targets endometrial cancer proliferation and survival
Source: Cell Mol Life Sci. 2022 Sep 19;79(10):524. doi: 10.1007/s00018-022-04541-6 (PMC9485191; doi:10.1007/s00018-022-04541-6)
Supplement: Supplementary file 2 — Supplementary file2 (DOCX 26 kb) [file 18_2022_4541_MOESM2_ESM.docx]

**Supplementary Materials and Methods and Tables**

***DNA constructs***

The pEBG2T vector encoding GST-tagged human ERK5 was previously described (1). The pCDNA3 vector encoding for SR-IkBα was from J.X. Comella (VHIR Barcelona, Spain; (2)). pGL4-AP1 (containing six copies of an AP1-response element fussed to luciferase gene) and PGLA4.32- NF-κB (five copies of a NF- κB response element) vectors were from Promega (Madison, WI, USA).

***shERK5 lentiviral production***

Stable and efficient silencing of endogenous ERK5 protein was achieved using lentivirus. Two pLKO.1 lentiviral vectors encoding for specific shRNA for two human MAPK7 mRNA sequences were used (TRCN0000010262/pLKO.1, seq. CCGGGCTGCCCTGCTCAAGTCTTTGCTCGAGCAAAGACTTGAGCAGGGCAGCTTTTT; TRCN0000010275/pLKO.1, seq. CCGGGCCAAGTACCATGATCCTGATCTCGAGATCAGGATCATGGTACTTGGCTTTTT; Sigma). Lentiviral particles were generated in HEK-293 cells by co-transfecting the virion vectors (psPAX2 and pMD2G) and the pLKO.1 ERK5-shRNA vectors using. After 4h, the medium was replaced with fresh medium. Forty-eight h post-transfection, the medium containing the viral particles was collected, centrifuged, filtered to remove cell debris, and stored at -80°C until use.

***Gene silencing***

Validated siRNAs targeting NEMO were from Sigma (ref. EHU032271) and ThermoFisher (ref. AM51331). Scrambled (control) siRNA was from ThermoFisher. siRNAs were transfected into cells using Lipofectamine-2000, following the manufacturer’s recommended protocol. The vectors encoding two different shRNAs targeting ERK5 were from Sigma (TRCN0000010262/pLKO.1; TRCN0000010275/pLKO.1). Lentiviral production is detailed at Supplementary Materials and Methods.

***In vitro ERK5 kinase activity assay***

A 40 μL reaction mixture was prepared containing 200 ng of pure active ERK5 (1) and the indicated amount of inhibitor in 50 mM Tris-HCl, pH 7.5, 0.1 mM EGTA, 1 mM 2-mercaptoethanol. Reactions were initiated by adding a mix of 10 mM magnesium acetate, 50 μM [γ^32^P]-ATP (400 cpm/pmol, Perkin-Elmer) and 200 μM PIMtide (ARKKRRHPSGPPTA) as substrates. Assays were carried out for 20 min at 30°C, terminated by applying the reaction mixture onto p81 paper, and the incorporated radioactivity measured by Cherenkov counting.

***Cell lysis and immunoblotting***

Cells were lysed in ice-cold RIPA buffer supplemented with 1 mM sodium-orthovanadate, 50 mM NaF and 5 mM sodium-pyrophosphate, sonicated and stored at −20°C. Subcellular fractionation was performed following standard procedures, as described before (1). Proteins were resolved in SDS-PAGE gels and electrotransferred onto a nitrocellulose membrane (Sigma-Aldrich). After incubation with the appropriated primary, detection was performed using horseradish peroxidase-conjugated secondary antibodies and enhanced chemiluminescence reagent (Bio-Rad). Primary antibodies used are given in **Supplementary Table S1**.

***Quantitative real-time PCR***

Total RNA was isolated from cells using RNeasy kit (Qiagen). cDNA was obtained using iScript™ cDNA Synthesis Kit (Bio-Rad). Real-time quantitative PCR assays were performed using TaqMan Gene Expression Master Mix and the probes human NEMO/IKBKG (Hs00415849_m1) and human GAPDH (Hs03929097_g1) (ThermoFisher Scientific). Amplifications were run in a Bio-Rad CFX96 real-time PCR system, using the following protocol: 50°C for 2 min, 95°C for 10 min, 39 cycles of 95°C for 15 s, 55°C for 1 min. Each value was normalized to GAPDH levels. Relative expression levels were determined using the 2^−ΔΔCt^ method. Real-time PCR was controlled by the Bio-Rad CFX Manager v 3.1 software.

***Gene reporter Luciferase assay***

Cells cultured in 12-well plates were transfected with 450 ng of NF-κB-driven (Stratagene) or AP 1-driven luciferase (Promega) reporter plasmids, and 50 ng Renilla luciferase (Promega). Luciferase activity assay was monitored using the dual luciferase kit (Promega), following the manufacturer’s instructions.

***Immunohistochemistry***

Samples from tumor xenografts were dissected, formalin-fixed and paraffin-embedded as described previously (3). Three μm-thickness sections were stained with haematoxylin and analyzed by immunohistochemistry using standard protocols. Briefly, deparaffinized samples were subjected to heat-induced antigen retrieval in citrate buffer. After blocking endogenous peroxidase with 3% H_2_O_2_, and reducing unspecific binding with and 5% goat serum and 0,1% Triton X-100, slices were then incubated with anti-cleaved caspase 3 antibody or anti-Ki67 antibody (Cell Signalling) and further processed with secondary antibody (LSAB2 horseradish peroxidase kit; Dako, Copenhagen, Denmark).

| **Primary antibody** | **Produced by** | **Supplied by** | **Concentration** |
| --- | --- | --- | --- |
| β-Actin | Mouse | Santa Cruz, sc-47778 | WB 1:5,000 |
| AKT | Rabbit | Cell Signalling, 9272 | WB 1:5,000 |
| Phospho-AKT (S473) | Rabbit | Cell Signalling, 9271 | WB 1:1,000 |
| Bak | Rabbit | Cell Signalling, 12105 | WB 1:1,000 |
| Bax | Mouse | BD Biosciences, 556467 | WB 1:1,000 |
| Bcl-2 | Mouse | Cell Signalling, 15071 | WB 1:1,000 |
| Bim | Rabbit | Cell Signalling, 2933 | WB 1:1,000 |
| BrdU | Mouse | BD Pharmingen | IF 1:200 |
| Cleaved caspase-3 | Rabbit | Cell Signalling, 9661 | WB 1:500  IHC 1:200 |
| Cleaved PARP | Rabbit | Cell Signalling, 5625 | WB 1:1,000 |
| CREB-1 | Mouse | Santa Cruz, sc-186 | WB 1:200 |
| ERK1/2 | Rabbit | Cell Signalling, 4695 | WB 1:8,000 |
| Phospho-ERK1/2 | Rabbit | Cell Signalling, 4376 | WB 1:5,000 |
| ERK5 | Rabbit | Cell Signalling, 3372 | WB 1:1,000 |
| GAPDH | Mouse | Invitrogen, AM4300 | WB 1:100,000 |
| GST | Rabbit | Santa Cruz, sc-459 | IC 1:500 |
| Hsp90-β | Rabbit | Invitrogen, PA3-012 | WB 1:10,000 |
| IKK-α | Mouse | Cell Signalling, 11930 | WB 1:1,000 |
| IKK-β | Rabbit | Cell Signalling, 8943P | WB 1:1,000 |
| IKK-$\gamma$/NEMO | Mouse | Cell Signalling, 2695 | WB 1:1,000 |
| IKB-α | Mouse | Cell Signalling, 4814 | WB 1:1,000 |
| JNK | Rabbit | Cell Signalling, 9252 | WB 1:1,000 |
| Phospho-JNK | Rabbit | Cell Signalling, 4668 | WB 1:1,000 |
| Ki67 | Rabbit | Cell Signalling, 5365 | IHC 1:600 |
| MEK5 (E-3) | Mouse | Santa Cruz, sc-365198 | WB 1:250 |
| p65 | Rabbit | Cell Signalling, 8242 | WB 1:1,000 |
| Phospho-p65 | Rabbit | Cell Signalling, 3033 | WB 1:1,000 |
| S6 | Rabbit | Cell Signalling, 2217 | WB 1:20,000 |
| Phospho-S6 | Rabbit | Cell Signalling, 4858 | WB 1:40,000 |

**Supplementary Table1.** List of antibodies used.

**References.**

1. Erazo T, Moreno A, Ruiz-Babot G, Rodríguez-Asiain A, Morrice NA, Espadamala J, et al. Canonical and kinase activity-independent mechanisms for extracellular signal-regulated kinase 5 (ERK5) nuclear translocation require dissociation of Hsp90 from the ERK5-Cdc37 complex. Mol Cell Biol. 2013;33:1671–86.

2. Marques-Fernandez F, Planells-Ferrer L, Gozzelino R, Galenkamp KMO, Reix S, Llecha-Cano N, et al. TNFα induces survival through the FLIP-L-dependent activation of the MAPK/ERK pathway. Cell Death Dis. 2013;4:e493–e493.

3. Erazo T, Lorente M, Lopez-Plana A, Munoz-Guardiola P, Fernandez-Nogueira P, Garcia-Martinez JA, et al. The New Antitumor Drug ABTL0812 Inhibits the Akt/mTORC1 Axis by Upregulating Tribbles-3 Pseudokinase. Clin Cancer Res. 2016;22:2508–19.
